# Berberine Alleviates Insulin Resistance and Inflammation *via* Inhibiting the LTB4–BLT1 Axis

**DOI:** 10.3389/fphar.2021.722360

**Published:** 2021-11-04

**Authors:** Minmin Gong, Huiyan Duan, Fan Wu, Yanlin Ren, Jing Gong, Lijun Xu, Fuer Lu, Dingkun Wang

**Affiliations:** ^1^ Institute of Integrated Traditional Chinese and Western Medicine, Tongji Hospital, Tongji Medical College, Huazhong University of Science and Technology, Wuhan, China; ^2^ Grade 2019 of Clinical Medicine, Medical College of China Three Gorges University, Hubei Yichang, China; ^3^ Department of Traditional Chinese Medicine, ZhongShan Hospital of Hubei Province, Wuhan, China; ^4^ Department of Integrated Traditional Chinese and Western Medicine, Tongji Hospital, Tongji Medical College, Huazhong University of Science and Technology, Wuhan, China

**Keywords:** leukotriene B4, insulin resistance, inflammation, berberine, diabetes

## Abstract

**Background:** Chronic low-grade inflammation is recognized as a key pathophysiological mechanism of insulin resistance. Leukotriene B4 (LTB4), a molecule derived from arachidonic acid, is a potent neutrophil chemoattractant. The excessive amount of LTB4 that is combined with its receptor BLT1 can cause chronic low-grade inflammation, aggravating insulin resistance. Berberine (BBR) has been shown to relieve insulin resistance due to its anti-inflammatory properties. However, it is not clear whether BBR could have any effects on the LTB4–BLT1 axis.

**Methods:** Using LTB4 to induce Raw264.7 and HepG2 cells, we investigated the effect of BBR on the LTB4–BLT1 axis in the progression of inflammation and insulin resistance.

**Results:** Upon exposure to LTB4, intracellular insulin resistance and inflammation increased in HepG2 cells, and chemotaxis and inflammation response increased in RAW264.7 cells. Interestingly, pretreatment with BBR partially blocked these changes. Our preliminary data show that BBR might act on BLT1, modulating the LTB4–BLT1 axis to alleviate insulin resistance and inflammation.

**Conclusions:** Our study demonstrated that BBR treatment could reduce intracellular insulin resistance and inflammation of hepatic cells, as well as chemotaxis of macrophages induced by LTB4. BBR might interact with BLT1 and alter the LTB4–BLT1 signaling pathway. This mechanism might be a novel anti-inflammatory and anti-diabetic function of BBR.

## Introduction

Leukotriene B4 (LTB4) is a proinflammatory lipid mediator, generated from arachidonic acid through the sequential steps of 5-lipoxygenase, 5-lipoxygenase-activating protein, and LTA4 hydrolase (Brandt et al., 2018). LTB4 is well-known to be a chemoattractant for leukocytes and mainly neutrophils. LTB4 interacts with its high-affinity receptor LTB4R1, also known as BLT1 (Saeki and Yokomizo, 2017). BLT1 is highly expressed in peripheral blood leukocytes and in lower amounts in the spleen, bone marrow, lymph nodes, and liver (Tager and Luster, 2003). LTB4 exerts its effects after binding to BLT1 to promote leukocyte infiltration into different tissues and regulate the production of pro-inflammatory cytokines. LTB4 is essential for a rapid response against infectious diseases, as it regulates the recruitment of leukocytes. However, excessive LTB4 can also lead to chronic inflammation, called “sterile inflammation”, triggered by the accumulation of metabolic products such as glucose, cholesterol, and free circulating fatty acids (He et al., 2020). Chronic inflammation accompanies many chronic diseases such as obesity, type 2 diabetes (T2DM), and atherosclerosis (Robbins et al., 2014).

Chronic low-grade inflammation is considered a critical pathophysiological mechanism of insulin resistance and T2DM (Muoio and Newgard, 2008). Macrophages are the primary sources of inflammatory mediators in the pathogenesis of T2DM (Lackey and Olefsky, 2016). Li et al. found that liver, muscle, and adipose tissue exhibited high LTB4 levels in obese high-fat diet (HFD)-fed mice, and LTB4 directly improved macrophage chemotaxis (Li et al., 2015). LTB4 was also previously shown to increase MyD88 expression and promote NF-κB p65 nuclear translocation and production of IL-6 and TNF-α in adipose tissue to enhance local inflammation in obese mice (Horrillo et al., 2010). LTB4 was also shown to aggravate insulin resistance *via* promoting inflammation-associated insulin resistance by recruiting macrophages, and reduce insulin sensitivity directly in muscle and liver by binding to BLT1 (Li et al., 2015). Reduction in LTB4 production and activity or blocking LTB4–BLT1 binding may reduce sterile inflammation and alleviate insulin resistance (Filgueiras et al., 2015). Therefore, the LTB4–BLT1 axis could be a new therapeutic target in the treatment of T2DM (Esmaili and George, 2015).

Berberine (BBR) is an isoquinoline alkaloid extracted from Chinese herbal medicines and widely used to treat diarrhea and diabetes (Pang et al., 2015). According to previous reports, BBR has broad pharmacological benefits, such as anti-inflammatory and hypolipidemic actions (Wei et al., 2016). BBR was found to inhibit the expression of inflammatory cytokines such as TNF-α and IL-1β in rats with T2DM, and decrease inflammation through the TLR4/MyD88/NF-κB signaling pathway (Gong et al., 2017). Given that LTB4 signaling modulated the MyD88/NF-κB pathway (Filgueiras et al., 2015), this study investigated if BBR could influence the LTB4–BLT1 axis to alleviate inflammation and insulin resistance.

## Methods

### Cell Culture

HepG2 cells (Kaiji, China) and RAW264.7 cells (Kaiji, China) were cultured in DMEM supplemented with 10% fetal bovine serum, 1% penicillin (100 units/ml), and streptomycin (100 mg/ml) at 37°C in a humidified incubator (5% CO_2_). Cells were subcultured when they reached a confluence rate of 70%. The day before the experiments, the cells were kept in DMEM containing 0.2% BSA for 12 h and then pretreated with 10 μM Berberine (Sigma-Aldrich, United States) or CP105696 (Sigma-Aldrich, United States) for subsequent testing. BBR and CP105696 were dissolved in DMSO at a concentration of 10 mM (×1,000 stock).

### LTB4 Preparation

LTB4 (Cayman Chemical, United States) was supplied as a solution in ethanol. The solution was further diluted in DMEM to a final concentration of 100 or 200 nM when used in experiments.

### Glucose Consumption

HepG2 cells were pre-incubated with DMEM containing 0.2% BSA for 12 h. After that time, the medium was replaced by DMEM containing BBR (10 µM) and LTB4 (100 nM) for 48 h. The medium was then removed and HepG2 cells were washed twice with PBS, and then incubated with 100 nM insulin (Macgene, China) for 30 min. Glucose concentrations of DMEM were determined using the glucose oxidase method (Nanjing Jiancheng, China). The amount of glucose consumption was calculated by subtracting the glucose concentrations of the blank wells from the remaining glucose in the cell-plated wells.

### Glucose Uptake Estimated

HepG2 cells (4 × 10^4^ cells/well) were cultured in a 24-well plate. The 48-h BBR treatment was followed by insulin treatment (100 nM) for 30 min and 0.1 mM 2-NBDG (Apexbio, United States) incubation for 45 min at 37°C. Images were obtained using identical acquisition settings on a fluorescence microscope (Nikon, Japan). Three independent replicates of the experiment were acquired.

### *In Vitro* Chemotaxis Assay

Chemotaxis assays were conducted with RAW264.7 cells in a modified Boyden transwell chamber (24-well plates, 8 μm pore size, Corning). 1 × 10^5^ RAW264.7 macrophages were placed in each upper chamber of the plate with or without BBR for 30 min; DMEM with or without LTB4 was placed in the lower chamber. After 4 h, cells migrated were fixed in formalin, and stained with Crystal Violet and counted. The migration index was calculated as follows: for each chamber, five non-overlapping fields under the microscope were chosen and counted. The mean number was calculated. The experiments were repeated three times.

### CCK-8 Assay

RAW264.7 and HepG2 cells were seeded in 96-well plates with a density of 10^4^ cells per well overnight and were set up using six complex holes in each group. BBR with a final concentration of 0.5, 1, 5, 10, 50, and 100 µM was added to each group. After 24 h of routine culture, 10 μl of CCK-8 solution was added to each well and cultured at 37°C for another 1 h. Cell proliferation index was calculated using a microplate reader, and the OD_450_ values were collected and analyzed.

### Western Blot Analysis

Protein concentrations were measured with a BCA protein assay kit. Using standard procedures, the protein samples (40 µg) were loaded on a SDS-PAGE gel, separated, and transferred to a polyvinylidene fluoride membrane. Membranes were then incubated with anti-AKT (#4685, CST, United States), p-AKT (#4060, CST, United States), Glut4 (#2213, CST, United States), IRS-1 (#3407, CST, United States), p-IRS-1 (#2381, CST, United States), JNK (#9252, CST, United States), *p*-JNK (#4668, CST, United States), IKKβ (#8943, CST, United States), *p*-IKKβ (#2696, CST, United States), NF-κB (#8242, CST, United States), p-NF-κB (#3033, CST, United States), BLT1 (#131041, Abcam, United kingdom), and GAPDH (#5174, CST, United States) antibodies. The GAPDH antibody was used as a loading control. The membranes were then co-incubated with fluorescent secondary antibodies. The Odyssey imaging system (LI-COR Biosciences, NE) was used to acquire the images and analyze protein expressions.

### Quantitative Real-Time PCR Analysis

RNA was extracted from cells using the Trizol reagent (Takara, Japan). The purity and concentration of the isolated RNA were measured with NanoDrop (Thermo, United States). A reverse transcriptase kit (Takara, Japan) was used to synthesize the cDNA. Quantitative analysis of mRNA expression was performed with a SYBR pre-mix EX TaqTM kit (Takara, Japan) in StepOne PCR detector (Applied Biosystems, United States). The relative amount of mRNA was expressed as 2^−ΔΔCT^. The primer sequences are listed in Table 1.

**TABLE 1:** Primers for RT-PCR assay.

Gene	Source	Forward (5′-3′)	Reverse (5′-3′)
IL-6	Mouse	TTC​TTG​GGA​CTG​ATG​CTG​GTG	GCC​ATT​GCA​CAA​CTC​TTT​TCT​C
TNF-α	Mouse	ACC​CTC​ACA​CTC​ACA​AAC​CA	ATA​GCA​AAT​CGG​CTG​ACG​GT
IL-1β	Mouse	TCA​AAT​CTC​GCA​GCA​GCA​CAT​C	CGT​CAC​ACA​CCA​GCA​GGT​TAT​C
CCl2	Mouse	ACC​AGC​AAG​ATG​ATC​CCA​ATG	GTG​CTT​GAG​GTG​GTT​GTG​GA
CCR2	Mouse	ACG​ATG​ATG​GTG​AGC​CTT​GTC	TGC​AGC​ATA​GTG​AGC​CCA​GA
BLT1	Mouse	CTG​TTG​CCC​ATT​GTT​CTA​CTG​TC	ATC​TCT​CTA​AAA​CTC​CAG​GTG​CC
CD11c	Mouse	AAA​GTG​GAG​CTC​GGC​AAG​AT	AAC​CAT​CTT​TGT​GTC​CTA​CCC​C
CD86	Mouse	TTG​GGC​ACA​GAG​AAA​CTT​GAT​AG	TTC​GGG​TGA​CCT​TGC​TTA​GAC
CD206	Mouse	CAG​GAG​GAC​TGC​GTG​GTT​ATG	GGT​TTG​CAT​CAG​TGA​AGG​TGG
GAPDH	Mouse	CCT​CGT​CCC​GTA​GAC​AAA​ATG	TGA​GGT​CAA​TGA​AGG​GGT​CGT
IL-6	Human	CAA​TGA​GGA​GAC​TTG​CCT​GGT​G	TGG​CAT​TTG​TGG​TTG​GGT​CA
IL-1β	Human	CGA​TCA​CTG​AAC​TGC​ACG​CTC	ACA​AAG​GAC​ATG​GAG​AAC​ACC​ACT​T
TNF-α	Human	GCT​GCA​CTT​TGG​AGT​GAT​CG	ATG​AGG​TAC​AGG​CCC​TCT​GA
CCL2	Human	GAT​CTC​AGT​GCA​GAG​GCT​CG	TTT​GCT​TGT​CCA​GGT​GGT​CC
MCP-1	Human	CTC​GCT​CAG​CCA​GAT​GCA​AT	CAC​TTG​CTG​CTG​GTG​ATT​CTT​CT
BLT1	Human	GCA​GGC​ATC​TGG​GTG​TTG​TC	CGA​CGC​CCT​ATG​TCC​GAG​T
GAPDH	Human	GGA​AGC​TTG​TCA​TCA​ATG​GAA​ATC	TGA​TGA​CCC​TTT​TGG​CTC​CC

### Statistical Analysis

GraphPad Prism software (GraphPad Software, CA) was used to analyze data. All the data were expressed as mean ± SD. Different groups were compared using one-way ANOVA following Tukey multiple comparison tests. *p*-values below 0.05 were considered statistically significant, and all tests were two-tailed.

## Results

### BBR Improved Insulin Resistance of HepG2 Cells Induced by LTB4

**FIGURE 1:**
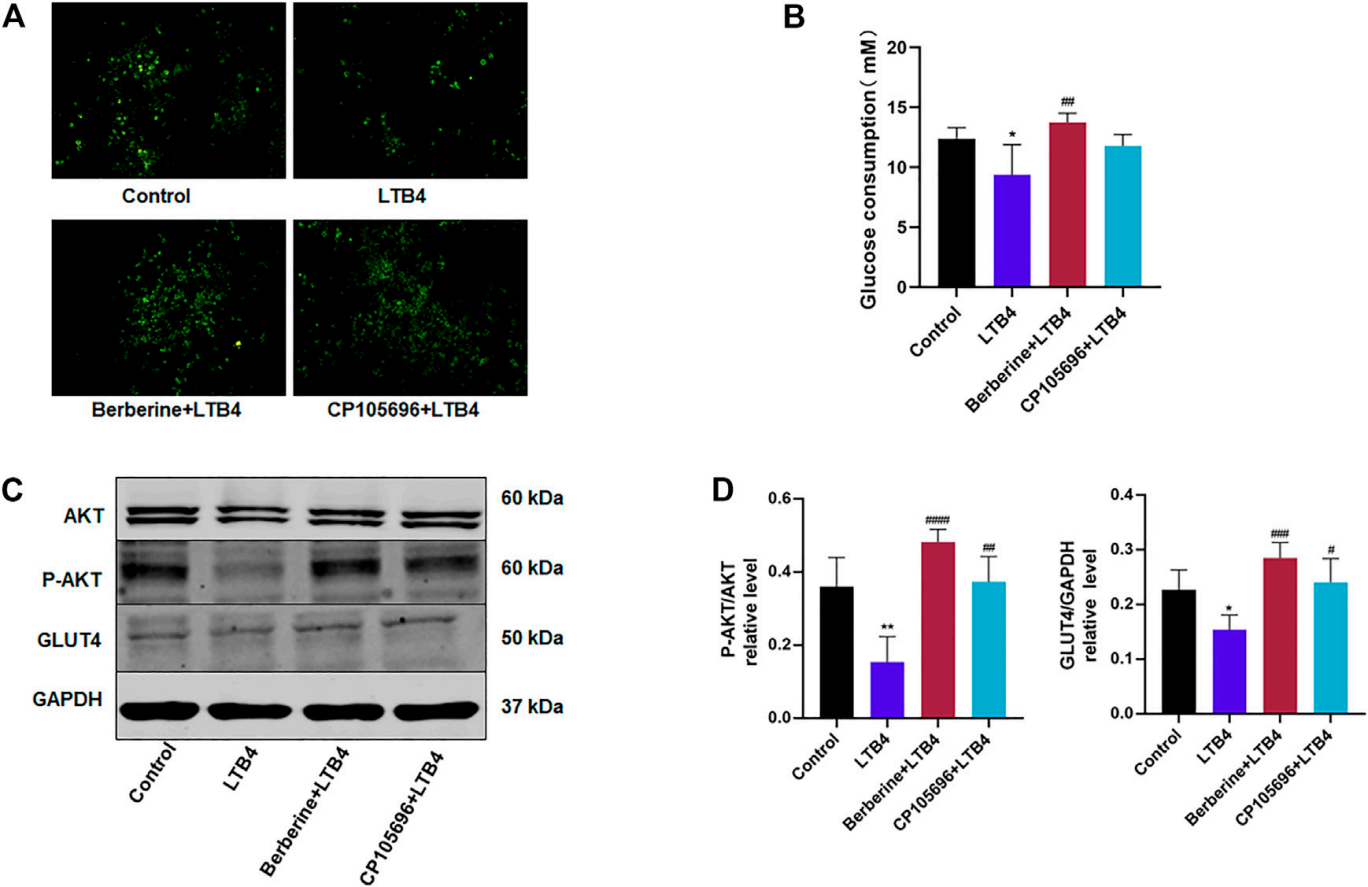
BBR improved insulin resistance of HepG2 cells induced by LTB4. **(A)** Glucose uptake of 2-NBDG assays from HepG2 cells detected by fluorescence microscopy. Scale bar, 200 μm (*n* = 3). **(B)** Glucose consumption was determined using the glucose oxidase method. The amount of glucose consumption was calculated by subtracting the glucose concentrations of the blank wells from the remaining glucose in the cell-plated wells (*n* = 5). **(C)** Western blots showed the protein levels of the insulin signaling pathway (P-AKT, GLUT4). **(D)** Densitometric analysis of *p*-AKT and GLUT4 in total protein was calculated (*n* = 4). Error bars represent mean ± SD. BBR, Berberine, 10 μM; CP105696, 10 μM; LTB4, Leukotriene B4, 100 nM ^*^ *p* < 0.05 vs. Control; ^#^ *p* < 0.05 vs. LTB4. ^**^ *p* < 0.01; ^##^ *p* < 0.01; ^###^ *p* < 0.001; ^####^ *p* < 0.0001.

Different concentrations of BBR were added to HepG2 cells for 24 h to determine the proper drug dosage for subsequent experiments. Cell survival was tested by a CCK-8 assay. Finally, a concentration of 10 µM was used, as this was the highest concentration without damage to cell proliferation (Supplementary Figure 1). CP105696, a pharmacological inhibitor of BLT1, was used as positive control (Showell et al., 1996). Li et al. previously showed that BLT1 was highly expressed in hepatocytes and directly led to intracellular insulin resistance (Li et al., 2015). Therefore, we tested whether LTB4 could cause insulin resistance in HepG2 cells. However, it was not sure that the concentration of LTB4, and mixed LTB4 with BBR or CP105696 had the cytotoxicity. We conducted another CCK-8 assay, and the result showed that LTB4 (100 nM), mixed LTB4 with BBR, or CP105696 had no effect on cell survival of HepG2 (Supplementary Figure 2). The preliminary result showed that LTB4 (100 nM) could decrease glucose uptake using 2-NBDG, while glucose uptake increased after 48 h of treatment with BBR or CP105696, the same as the control group. Quantified glucose consumption results verified this phenomenon (Figures 1A,B). Next, we detected changes in proteins in the signaling pathway (Muoio and Newgard, 2008). BBR and CP105696 promoted the expressions of p-AKT and Glut4, which were reduced in the LTB4 group (Figures 1C,D). These experiments suggested that LTB4 directly led to intracellular insulin resistance in HepG2 cells, and BBR notably reversed these changes.

### BBR Attenuated the Inflammation Response of HepG2 Cells Induced by LTB4

**FIGURE 2:**
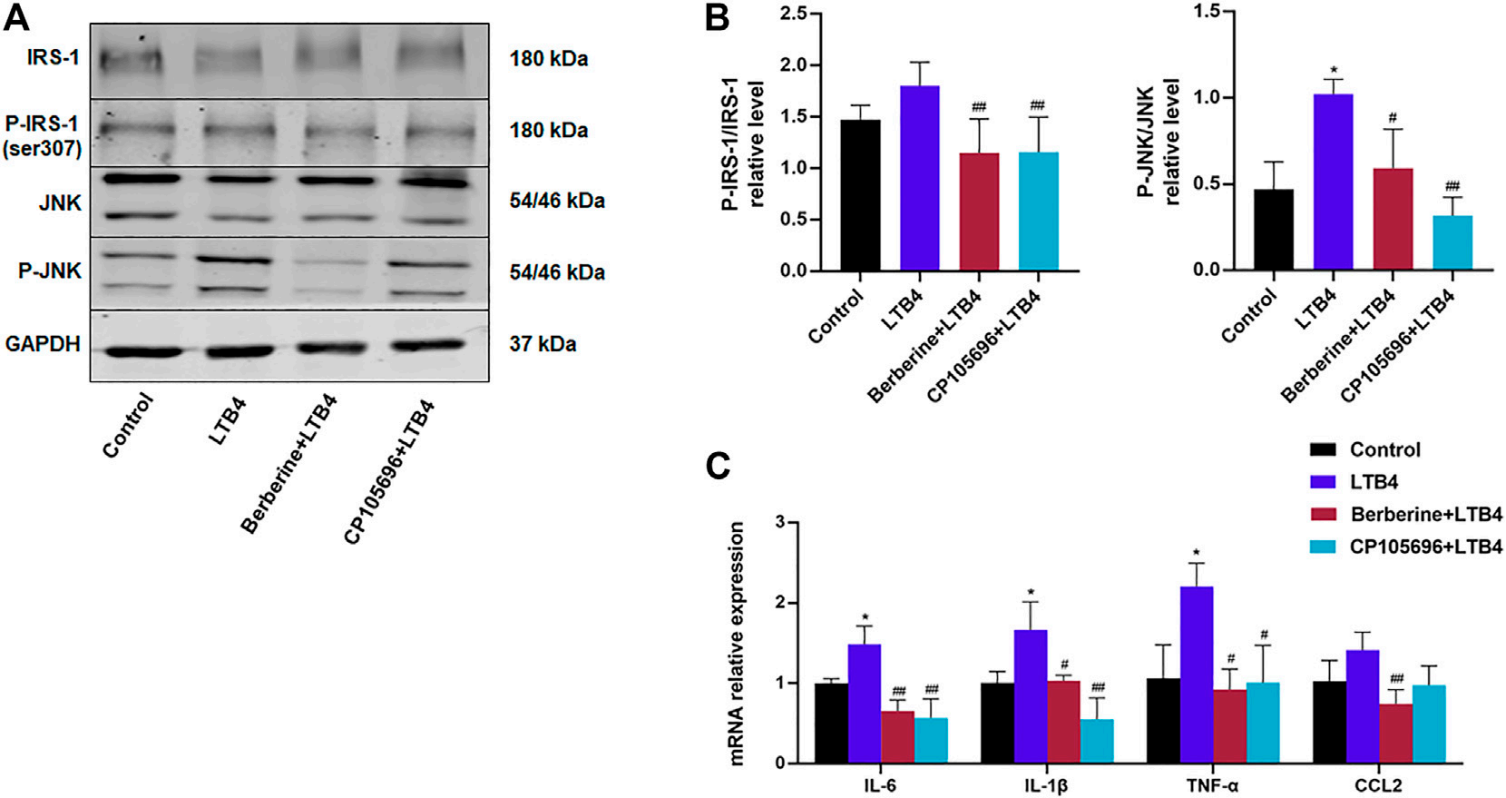
BBR attenuated the inflammation response of HepG2 cells induced by LTB4. **(A)** Western blots showed p-IRS-1 and *p*-JNK protein levels in HepG2 cells. **(B)** Densitometric analysis of p-IRS-1 and *p*-JNK in total protein was calculated (*n* = 3–5). **(C)** Gene expressions of IL-6, TNF-α, IL-1β, and CCL2 were determined by RT-PCR (*n* = 4). Error bars represent mean ± SD. BBR, Berberine, 10 μM; CP105696, 10 μM; LTB4, Leukotriene B4, 100 nM ^*^ *p* < 0.05 vs. Control; ^#^ *p* < 0.05 vs. LTB4. ^**^ *p* < 0.01; ^##^ *p* < 0.01.

It has been reported that LTB4 can recruit phagocytes and promote the production of inflammatory factors (Okamoto et al., 2010). However, it is not clear whether LTB4 could directly cause inflammation without the help of leukocytes. The phosphorylation of the insulin receptor substrate 1 (IRS-1) in Ser307, by p-JNK and p-IKKβ, can alter its downstream signaling (Muoio and Newgard, 2008). This was consistent with the results above showing that LTB4 decreased the protein level of p-IRS-1 (Figures 2A,B). The data showed that BBR or CP105696 reduced the inflammatory protein (*p*-JNK) in HepG2 cells compared to the LTB4 group (Figures 2A,B). In contrast, no differences were found in the *p*-IKKβ protein level (data not shown). In addition, the mRNA expression of inflammatory cytokines was examined further using qPCR. As shown in Figure 2C, cells in the LTB4-treated group exhibited higher IL-6, TNF-α, IL-1β, and CCL2. After treatment with BBR or CP105696, the inflammatory situation improved significantly. The mRNA expression of IL-6, TNF-α, IL-1β, and CCL2 decreased to the level of control group after treatment. These data indicated that LTB4 directly contributed to increasing inflammation cytokines and proteins that impaired the insulin signaling pathway, and BBR could disturb the function of LTB4.

### BBR Attenuated the Inflammation Response of RAW264.7 Cells Induced by LTB4

**FIGURE 3:**
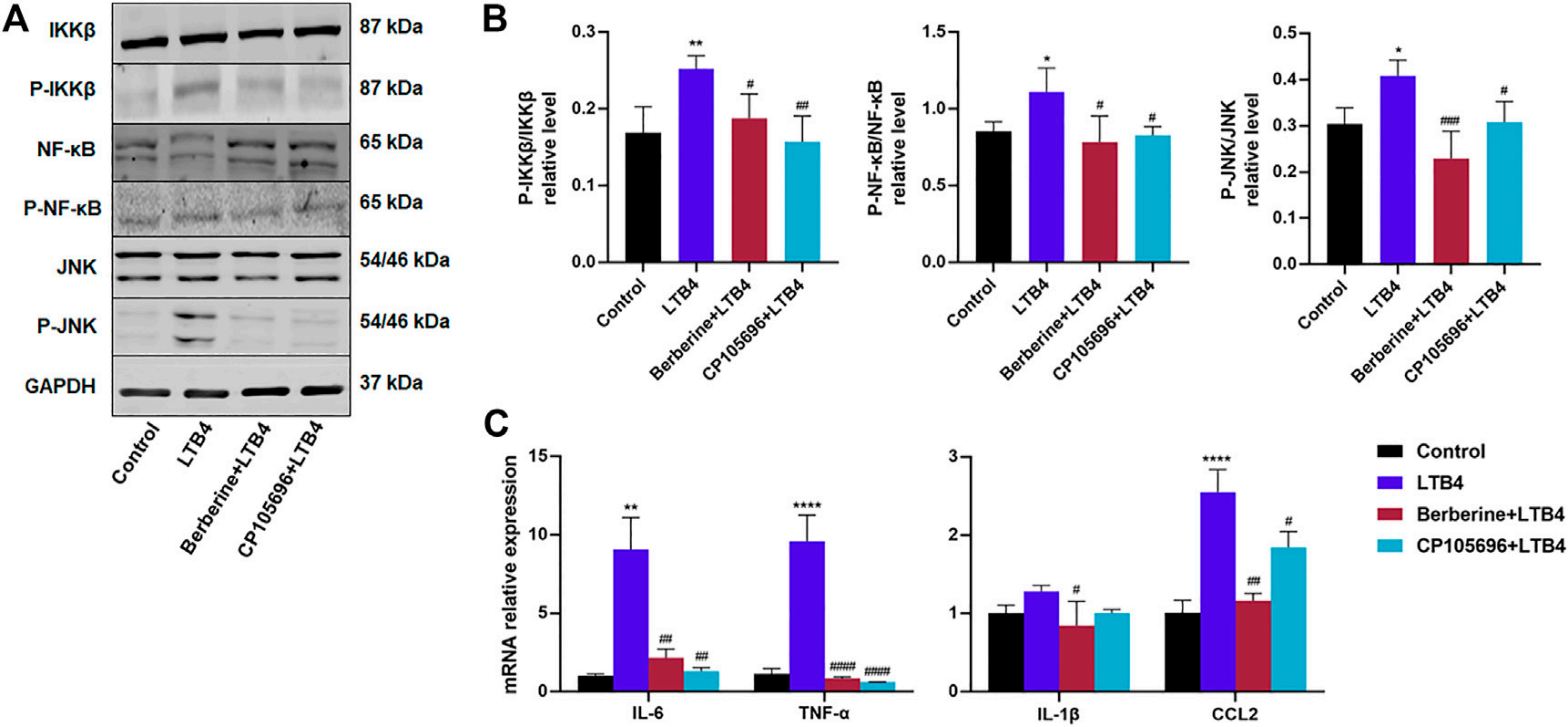
BBR attenuated the inflammation response of RAW264.7 cells induced by LTB4. **(A)** Western blots showed the protein levels of *p*-JNK, p-NF-κB, and *p*-IKKβ in RAW264.7 cells **(B)** Densitometric analysis of *p*-JNK, p-NF-κB, and *p*-IKKβ in total protein was calculated (*n* = 4). **(C)** Gene expressions of IL-6, TNF-α, IL-1β, and CCL2 were determined by RT-PCR (*n* = 3–4). Error bars represent mean ± SD. BBR, Berberine, 10 μM; CP105696, 10 μM; LTB4, Leukotriene B4, 200 nM ^*^ *p* < 0.05 vs. Control; ^#^ *p* < 0.05 vs. LTB4. ^**^ *p* < 0.01; ^##^ *p* < 0.01; ^###^ *p* < 0.001; ^####^ *p* < 0.0001.

LTB4 is a potent macrophage chemotaxis agent and the driving factor in chronic inflammation (Nagareddy et al., 2014). Therefore, RAW264.7 cells were chosen to investigate the effect of LTB4 on macrophages. As shown in Supplementary Figure 1, the cells were also treated with 10 µM BBR. The LTB4 (200 nM) and mixed LTB4 with BBR or CP105696 had no cytotoxicity on RAW264.7 cells (Supplementary Figure 2). LTB4 sharply increased the protein levels of p-JNK, p-NF-κB, and p-IKKβ (Figure 3A). The data suggested that the treatment of BBR or CP105696 for 24 h notably decreased the inflammatory protein expressions of p-JNK, p-NF-κB, and p-IKKβ (Figures 3A,B). Furthermore, we found that IL-6, TNF-α, IL-1β, and CCL2 mRNA levels increased by intervention with LTB4 (200 nM). However, BBR and CP105696 could improve this situation (Figure 3C). Collectively, BBR greatly attenuated intracellular inflammation of RAW264.7 cells induced by LTB4.

### BBR Suppressed Chemotaxis of LTB4-Activated RAW264.7 Cells and Might Inhibit Polarization of RAW264.7 Cells to M1 Macrophages

**FIGURE 4:**
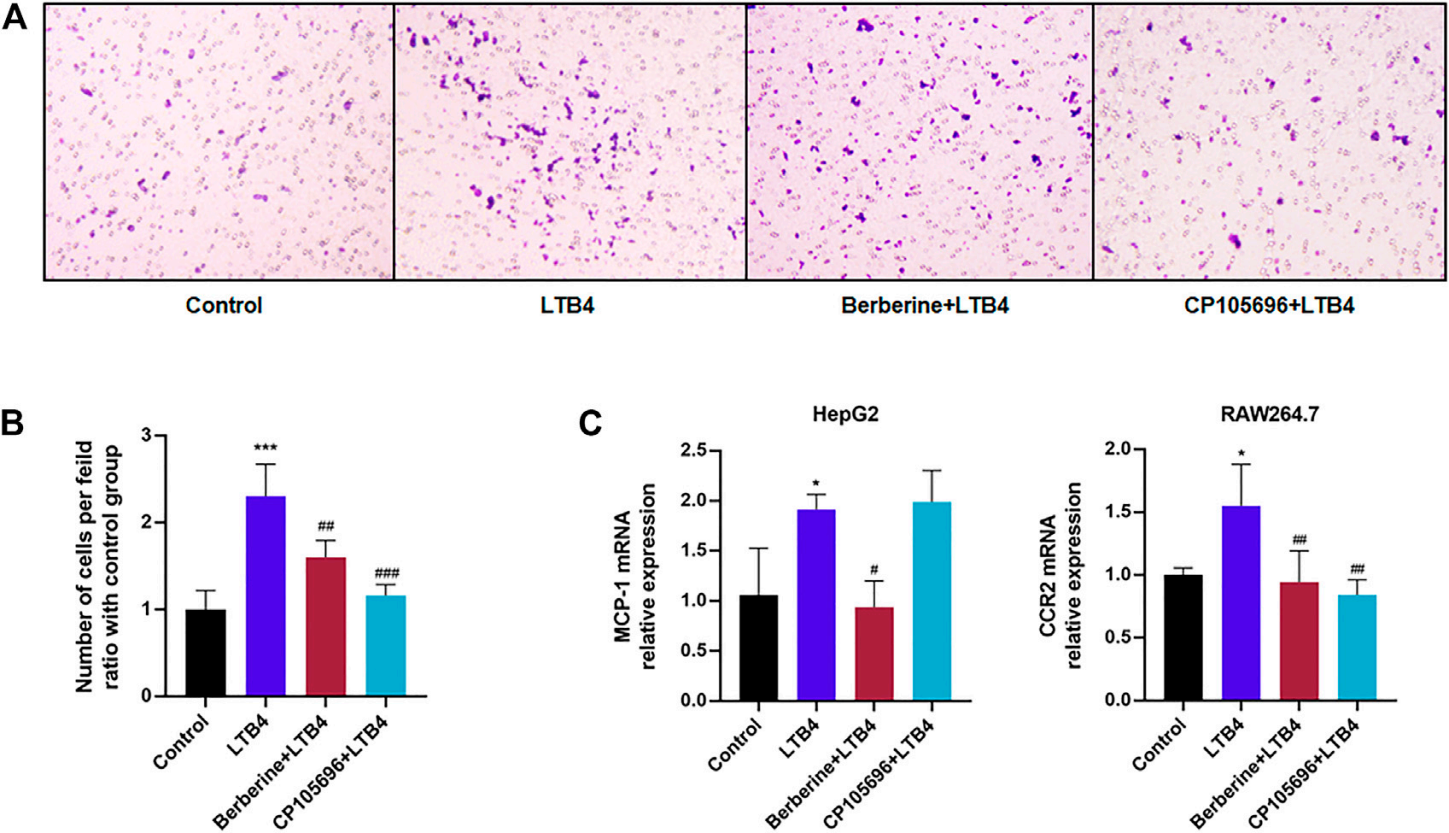
BBR suppressed chemotaxis of RAW264.7 cells activated by LTB4. **(A)** *In vitro* chemotaxis assays of RAW264.7 macrophages were performed. Scale bar, 200 μm. Cells were fixed in formalin and stained with crystal violet. **(B)** The quantified number of RAW264.7 that were stained in each group (*n* = 3). **(C)** Gene expressions of MCP-1 in HepG2 cells and CCR2 in RAW264.7 cells were analyzed by RT-PCR (*n* = 4). Error bars represent mean ± SD. BBR, Berberine, 10 μM; CP105696, 10 μM; LTB4, Leukotriene B4, 200 nM ^*^ *p* < 0.05 vs. Control; ^#^ *p* < 0.05 vs. LTB4. ^***^ *p* < 0.01; ^##^ *p* < 0.01; ^###^ *p* < 0.001.

**FIGURE 5:**
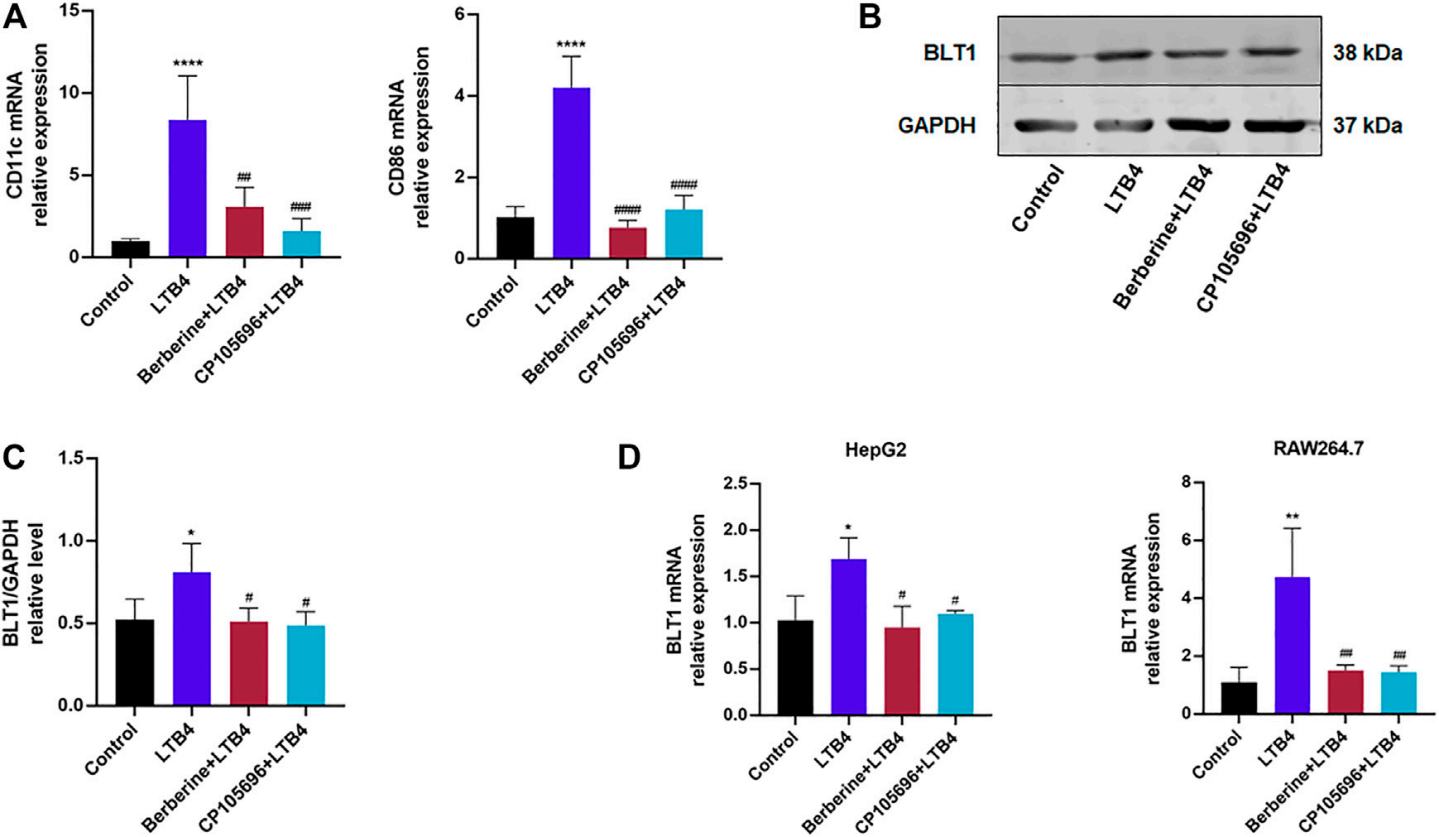
BBR may inhibit LTB4-induced polarization of RAW264.7 cells to M1 macrophages and regulate BLT1 of HepG2 and RAW264.7 cells. **(A)** Gene expressions of CD11c and CD86 in RAW264.7 were determined by RT-PCR (*n* = 4). **(B)** Western blots exhibited the protein level of BLT1 in HepG2 cells. **(C)** Densitometric analysis of BLT1 in the total protein of HepG2 cells was calculated (*n* = 4). **(D)** BLT1 gene expression in HepG2 and RAW264.7 cells determined by RT-PCR (*n* = 3). Error bars represent mean ± SD. Berberine, 10 μM; CP105696, 10 μM; LTB4, Leukotriene B4. *p < 0.05 vs. Control; #*p* < 0.05 vs. LTB4. **p < 0.01; ***p < 0.001, ****p < 0.0001; ##*p* < 0.01; ###*p* < 0.001; ###*p* < 0.0001.

To further elucidate if BBR could act on the LTB4–BLT1 axis, we conducted an *in vitro* chemotaxis assay. As illustrated in Figures 4A,B, BBR and CP105696 downregulated chemotaxis of RAW264.7 activated by LTB4. Morinaga’s research indicated that the chemokine receptor type 2 (CCR2) was highly expressed in recruited hepatic macrophages in obese mice (Morinaga et al., 2015). Figure 4C showed that LTB4 significantly increased CCR2 mRNA expression. Furthermore, the expression of MCP-1, the ligand of CCR2, was upregulated in HepG2 cells induced by LTB4. CD11c and CD86 mRNA expression levels, hallmarks of M1-type macrophages (Lumeng et al., 2007), increased sharply in RAW264.7 cells after with LTB4 treatment (Figure 5A). Inversely, BBR lowered the expression of MCP-1 and CCR2 (Figure 4C). Taken together, our results demonstrated that BBR reduced chemotaxis and polarization of macrophages induced by LTB4.

### BBR May Regulate BLT1 of HepG2 and RAW264.7 Cells

Given that BLT1 was the high-affinity receptor for LTB4, we hypothesized that BBR could affect BLT1 expression to inhibit the LTB4–BLT1 axis. As preliminarily shown in Figures 5B,C, the protein expression of BLT1 was downregulated by the treatment of BBR in HepG2 cells. Moreover, BBR decreased BLT1 mRNA expression levels in HepG2 and RAW264.7 cells (Figure 5D). These data implied that BBR might influence BLT1.

## Discussion

In the current study, BBR alleviated LTB4-induced intracellular insulin resistance and inflammation of hepatic cells. Besides, our results revealed that BBR reduced the chemotaxis and inflammation response of macrophages activated by LTB4. The possible mechanism might be its effect on the LTB4–BLT1 axis that was viewed as a new target to treat metabolic diseases (Johnson et al., 2017).

Insulin resistance is a complicated pathophysiologic mechanism characterized by impaired insulin signaling in insulin-sensitive tissues. Inflammatory mediators such as cytokines and other factors participate in the pathological process, and other mechanisms, such as lipotoxicity, can also contribute to insulin resistance (Samuel et al., 2010). In visceral fat from obese subjects, macrophages can account for a large proportion of the total cells and secrete a variety of factors that could aggravate inflammation. In addition, other immune cell types, such as lymphocytes, B2 cells, and neutrophils also contribute to the tissue inflammatory state in obesity and diabetes (Yang et al., 2010; Ying et al., 2017). LTB4 is a kind of proinflammatory lipid mediator. It bounds with high affinity to BLT1, G protein-coupled receptor. After specifically binding to BLT1, LTB4 exerts robust effects on promoting leukocyte infiltration into various tissues and regulates pro-inflammatory cytokine production. On the basis of current studies, the attempts to inhibit the LTB4–BLT1 system might be more efficacious, even that this modality cannot treat insulin resistance directly.

Given that there was no expression of BLT1 on adipocytes, hepatic cells were chosen as the research object. Li’s research firstly pointed out that LTB4 can directly affect hepatocytes and myocytes to impair insulin signaling (Li et al., 2015). This phenomenon was testified in our study. LTB4 impaired the insulin signals of HepG2 cells, manifesting reduced glucose uptake and reduced p-IRS-1, p-AKT, and GlUT4 expression. P-IRS-1 was known to be phosphorylated by p-JNK, that LTB4 also increased. Stimulated production of LTB4 by primary tissue cells such as lymphocytes or other resident tissue cell types could be an early trigger for the chronic tissue inflammatory state in obesity and diabetes. Our results showed that LTB4-induced inflammation exacerbated intracellular insulin resistance. Many previous studies showed that LTB4 had a potent ability of leukocyte chemotaxis, such as neutrophils and macrophages. Then, macrophages secreted inflammatory factors that aggravated sterile inflammation (Saeki and Yokomizo, 2017). In general, LTB4 triggered and stabilized inflammation with the help of recruited leukocytes. However, our research suggested that LTB4 might directly upregulate inflammation cytokines of hepatic cells that could influence the intracellular environment. The results indicated that BBR and CP105696 pretreatment could partially reverse the symptoms mentioned above. Thus, BBR may improve insulin resistance and decrease intracellular inflammation cytokines through the LTB4–BLT1 axis.

To further explore our hypotheses, we intervened macrophages, RAW264.7, with LTB4 and treated them with BBR. As illustrated in the results, BBR could partially block the LTB4-induced chemotaxis on macrophages. One previous research indicated that BBR inhibited M1 macrophage activation in adipose tissue (Ye et al., 2016). Consistent with the result above, BBR could reduce gene expression in M1 macrophages, which were markedly upregulated by LTB4. Taken together, BBR may react on the LTB4–BLT1 axis to disturb the chemotaxis and polarization of macrophages.

BBR is an isoquinoline alkaloid found in some medicinal plants, including *Coptis chinensis* (Franch) and *Phellodendron chinense* (Schneid). BBR has been widely researched in the treatment of insulin resistance and diabetes, and manifested its powerful hypoglycemic and hypolipidemic functions with multiple pathways and targets (Pirillo and Catapano, 2015). Recent studies showed that BBR decreased hyperglycemia, alleviated insulin resistance, and inhibited lipid synthesis possibly *via* the activation of adenosine monophosphate-activated protein kinase (AMPK) (Turner et al., 2008). Our previous study demonstrated that the MyD88/NF-κB pathway was the target inflammation pathway of BBR (Gong et al., 2019). The MyD88/NF-κB pathway was also known as the downstream pathway of the LTB4 pathway (Sánchez-Galán et al., 2009), which was verified in our study. BBR negatively regulated the expression of the p-NF-κB protein in macrophages caused by LTB4. Combined with the results above, BBR can regulate the LTB4 pathway.

The surprising function of LTB4 shown in our data on the insulin signaling pathway and inflammation may be due to the well-expressed BLT1 on hepatic cells and macrophages. BLT1 inhibition can block chemotaxis and tracking of macrophages and other immune cells into metabolic tissue, inhibiting the ultimate inflammation–insulin resistance syndrome. This was consistent with previous studies in BLT1-KO mice, which showed an anti-inflammatory phenotype along with improved glucose tolerance and insulin sensitivity (Spite et al., 2011). Given that BBR had great effect as well as CP105696, we attempted to find out another exact site of BBR on the LTB4–BLT1 axis. BLT1 protein and mRNA levels were detected in two types of cells. The preliminary results implied that BBR might impact BLT1. However, it needed more further research.

Chronic low-grade inflammation, particularly in adipose tissue and liver, played an essential role in obesity-induced insulin resistance and glucose intolerance. In obese or diabetic mice, macrophages were attracted and accumulated in adipose tissue and liver (Oh et al., 2012; Li et al., 2015). Recruited hepatic macrophages were recently shown to represent a sizable liver macrophage population in the context of obesity, and they mainly enhanced the severity of obesity-induced inflammation and hepatic insulin resistance compared to Kupffer cells (Morinaga et al., 2015). LTB4 was increased dramatically in the liver of obese mice (Li et al., 2015) and played an essential role in recruiting macrophages. BBR treatment inhibited chemotaxis of macrophages to improve inflammation-associated insulin resistance as well as promoted insulin resistance directly. BBR has been demonstrated to alleviate insulin resistance and diabetes *via* multiple pathways. Inhibition of the LTB4–BLT1 axis may become a new target for BBR. In summary, our study provided evidence that BBR could affect the LTB4–BLT1 axis and might interact with BLT1 to alleviate insulin resistance and inflammation.

## Data Availability

The original contributions presented in the study are included in the article/Supplementary Material, further inquiries can be directed to the corresponding authors.
