# Supplementary material for: Berberine Alleviates Insulin Resistance and Inflammation via Inhibiting the LTB4–BLT1 Axis
Source: Front Pharmacol. 2021 Nov 4;12:722360. doi: 10.3389/fphar.2021.722360 (PMC8599302; doi:10.3389/fphar.2021.722360)
Supplement: Supplementary file 1 [file DataSheet1.PDF]

Supplementary materials

Figure 1.

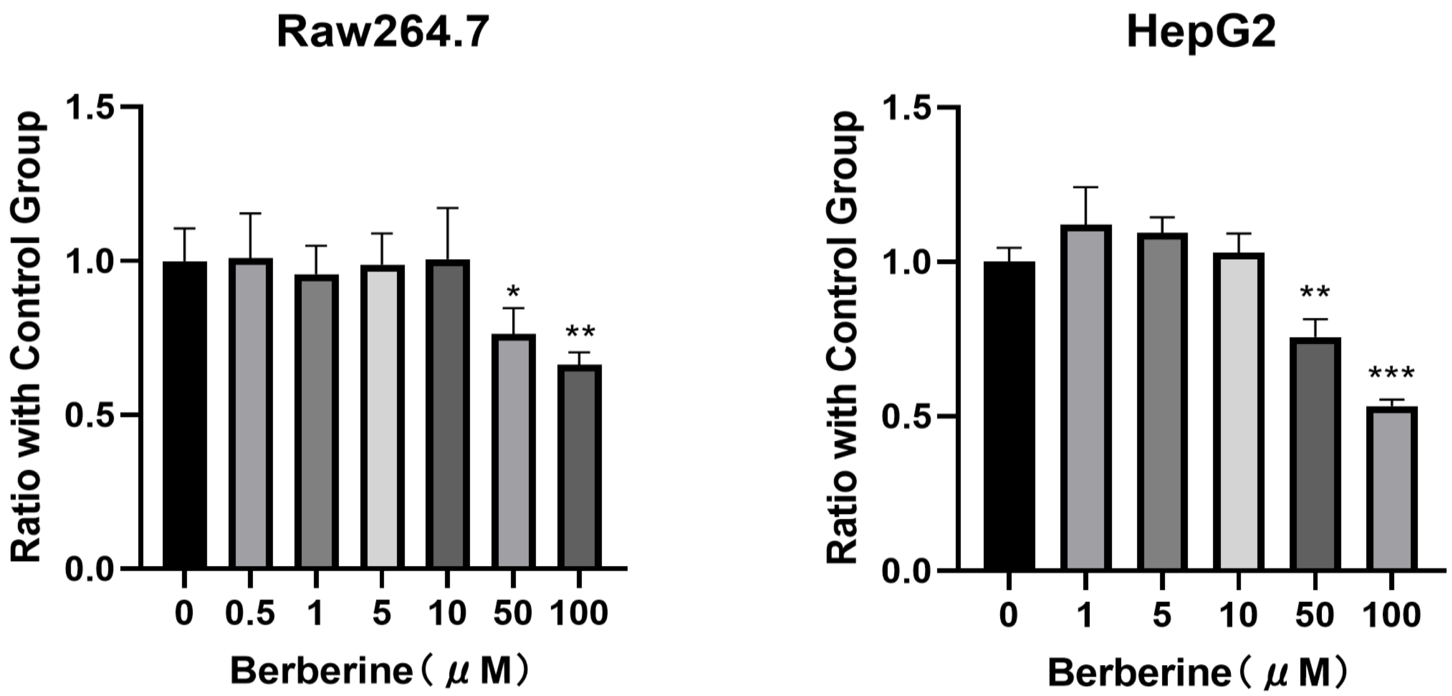

Figure 1.The effects of Berberine with different concentrations on cell proliferation of Raw 264.7 and HepG2 cells. Error bars represent mean ± SD (n=4). $^{*}P$ <0.05 vs. Control; $^{**}P$ < 0.01; $^{***}P$ < 0.001.

Figure 2.

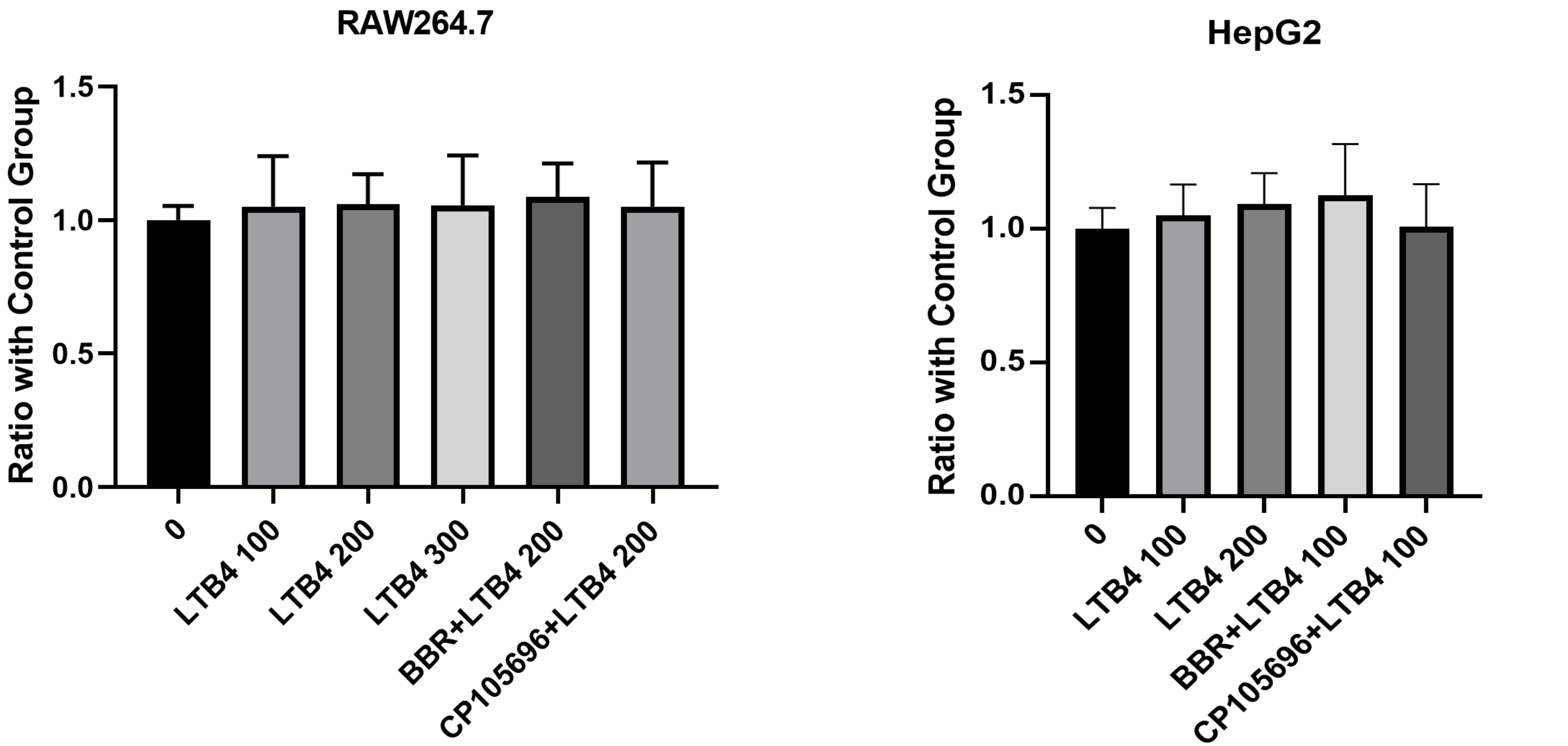

Figure 2.The effects of different treatments on cell proliferation of Raw 264.7 and HepG2 cells.
Error bars represent mean ± SD (n=7). LTB4 100, Leukotriene B4 100 nM; LTB4 200, Leukotriene B4 200 nM; LTB4 300, Leukotriene B4 300 nM. BBR, Berberine, 10 μM; CP105696, 10 μM.
